# miR-16-1 Promotes the Aberrant *α*-Synuclein Accumulation in Parkinson Disease via Targeting Heat Shock Protein 70

**DOI:** 10.1155/2014/938348

**Published:** 2014-06-23

**Authors:** Zhelin Zhang, Yan Cheng

**Affiliations:** ^1^Department of Neurology, General Hospital of Tianjin Medical University, No. 154, Anshan road, Heping District, Tianjin 300071, China; ^2^Department of Neurology, Affiliated Hospital of Inner Mongolia Medical University, No. 1 Tongdao North Street, Hohhot, Mongolia 010059, China

## Abstract

There is striking evidence that heat shock protein 70 (Hsp70) negatively regulates *α*-synuclein aggregation, which plays a significant role in the formation and progression of Parkinson disease (PD). However, how the Hsp70 in neurons fails to prevent or even reverse *α*-synuclein aggregation and toxicity in PD still remains to be determined. In the present study, we constructed an *α*-synuclein-overexpressed human neuroblastoma cell line, SH-SY5Y-Syn, in which the blockage of Hsp70 promoted *α*-synuclein aggregation. And we also found that miR-16-1 downregulated Hsp70 and promoted *α*-synuclein aggregation in the SH-SY5Y-Syn cells. This study revealed a novel regulatory mechanism of Hsp70 expression, which might contribute to the PD development.

## 1. Introduction

Parkinson disease (PD) is the second most common neurodegenerative disorder after Alzheimer disease [1], prominent characteristics of which include the degeneration of dopaminergic cells within the substantia nigra pars compacta (SNpc) [2] and the aberrant intracellular protein aggregation including, but not limited to, *α*-synuclein [3, 4]. Although how *α*-synuclein induces cytotoxicity still remains to be determined, there is striking evidence that *α*-synuclein plays a significant role in the formation and progression of PD [5, 6].

*α*-Synuclein is abundantly expressed in the nervous system [7], during neuronal development [8], and modulated in conditions that alter plasticity or confer injury [8, 9]. And the key role in PD of the aberrant *α*-synuclein aggregation has been underlined by the nontoxicity of the nonaggregated *α*-synuclein [10] and by the assistance of overexpressing molecular chaperones, such as heat shock proteins (HSPs) in refolding of aggregated *α*-synuclein [11]. Therefore, the function defect of HSPs is believed to play a key role in the aberrant *α*-synuclein aggregation of PD [11, 12]. Hsp70 is the most studied molecular chaperone, linking to PD and *α*-synuclein aggregation. Studies have demonstrated the negative regulatory role of Hsp70 in *α*-synuclein aggregation in PD and in *α*-synuclein-induced toxicity in cells [12, 13]. Obviously, despite being the prominent defense mechanism, the Hsp70 system in neurons fails to halt, prevent, or even reverse *α*-synuclein misfolding and toxicity in PD.

MicroRNAs (miRNAs) are endogenous noncoding RNAs (18–22 nt) that regulate gene expression [14] in diverse cell processes in mammals [15]. Recently, miRNAs have been suggested to play important roles in brain functions [16]. Some specifically expressed or enriched miRNAs in the brain have been confirmed to associate with memory, neuronal differentiation, and synaptic plasticity [17]. And the role of miRNAs in neurodegeneration has also been suggested in several reports [18–20]. Furthermore, there are miRNAs being reported to play a role in the *α*-synuclein aggregation in PD [21, 22]. Here, we show that miR-16-1 promotes the aberrant *α*-synuclein accumulation via targeting heat shock protein 70 in human neuroblastoma cell line SH-SY5Y.

## 2. Materials and Methods

### 2.1. Reagents, Cell Culture, and Treatment

MAL3-101, an allosteric Hsp70 modulator, inhibiting Hsp70 ATPase activity [23] was purchased from the A Chemtek Inc. (Worcester, MA, USA). SH-SY5Y (human neuroblastoma cell line) was purchased from ATCC. Cells were cultured in Dulbecco's modified Eagle's medium (DMEM) (Invitrogen, Carlsbad, CA, USA) containing 10% FBS (Invitrogen, Carlsbad, CA, USA) or maintained in DMEM supplemented with 2% FBS. To generate an *α*-synuclein overexpressed cell line, the wild *α*-synuclein coding sequence was amplified and cloned into the pcDNA3.1(+) vector. And SH-SY5Y cells were transfected with the *α*-Syn-pcDNA3.1(+) or control pcDNA3.1(+) vectors and were selected in the presence of 1.5 mg/mL G418. The 20 or 40 nM Hsp70 siRNA (Sangon, Shanghai, China) was transfected with Lipofectamine 2000 (Invitrogen, Carlsbad, CA, USA) into the SH-SY5Y cells to abrogate the Hsp70 expression. The miR-16-1 mimics or miRNA control (GenePharma, Shanghai, China) with 25 or 50 nM was also transfected with Lipofectamine 2000.

### 2.2. RNA Isolation, Reverse Transcription, and RT-qPCR

Total cellular RNA was isolated with PureLink RNA Minikit (Invitrogen, Carlsbad, CA, USA), and miRNAs were isolated using mirVana miRNA Isolation Kit (Ambion, Austin, TX, USA) according to manuals. The expression of *α*-Syn and Hsp70 in mRNA level or of miR-16-1 was quantified by the real-time RT-PCR method with Takara One-Step RT-PCT kit (TaKaRa Bio Inc., Tokyo, Japan). mRNA samples were amplified using primer sets specific for the genes of interest on a Lightcycler 480 II (Roche, Diagnostics, GmbH, Germany). Relative quantification was determined using the ΔΔCt method using *β*-actin as reference gene [24].

### 2.3. Western Blot Analysis

Approximately 10^5^ cells were lysed with the cytoplasmic protein extraction Kit (ZmTech Scientific Inc.) and quantified using Bradford Reagent (Bio-Rad, Hercules, CA, USA); protein samples were separated by a 12% gradient SDS-PAGE gel, transferred to PVDF membrane, and blocked in 5% skimmed milk. Rabbit polyclonal antibodies to *α*-Syn, Hsp70, or *β*-actin (1 : 300 to 1000) (Sigma-Aldrich, St. Louis, MO, USA) were used to quantify the molecular expression, with ECL detection systems (Pierce, Rockford, IL,USA).

### 2.4. Immunocytochemistry

Cells were cultured on polylysine-coated coverslips for 24 h followed by treatment with various reagents and/or transfection, after fixation in 4% paraformaldehyde, and permeabilized with 0.25% Triton X-100. After being blocked with 1% normal goat serum, 20 mg/mL BSA, and 0.25% Triton X-100 in PBS, cells were incubated firstly with primary antibodies for *α*-Syn (Sigma-Aldrich, St. Louis, MO, USA) and secondly with anti-rabbit Alexa Fluor 488-conjugated secondary antibody (Danvers) and were analyzed on a fluorescence microscope (Axiovert 200M, Zeiss, Germany). And the *α*-Syn aggregation dots were counted with Image J software.

### 2.5. Statistical Evaluation

For the analysis of *α*-Syn, Hsp70, and miR-16-1 expression and the analysis of *α*-Syn aggregation dots between two groups, statistical evaluations are presented as mean ± SE. Data were analyzed using the Student's* t*-test, and a statistical significance was considered when *P* < 0.05.

## 3. Results

### 3.1. Construction of SH-SY5Y Cells Overexpressing *α*-Synuclein

**Figure 1:**
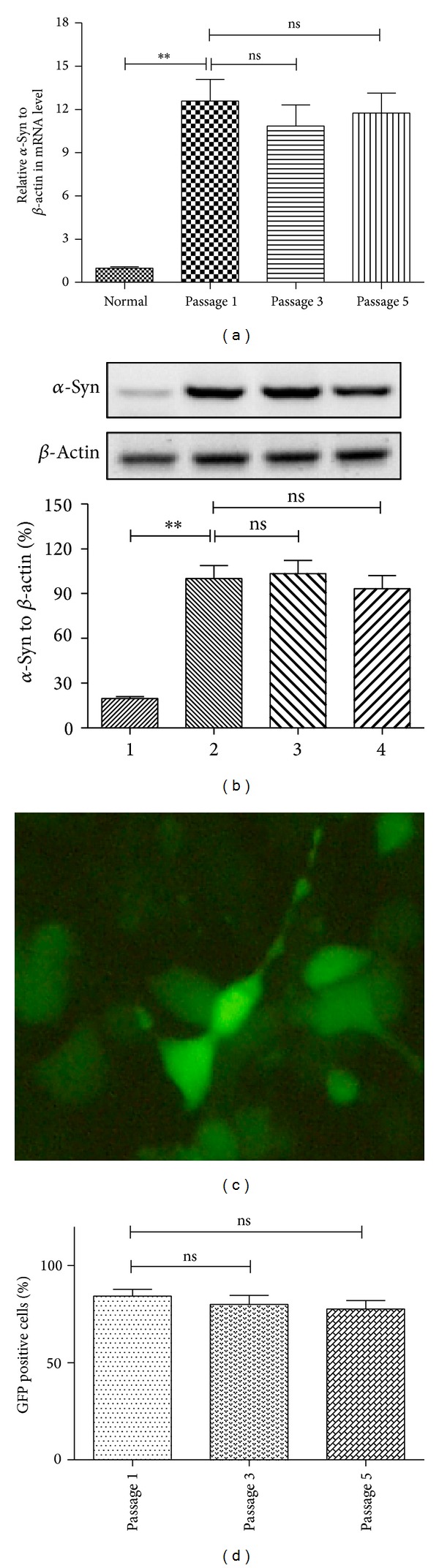
*α*-Synuclein is overexpressed in SH-SY5Y-Syn cells. (a) RT-qPCR analysis of *α*-synuclein overexpression in SH-SY5Y-Syn cell lines after serial passages, compared to *β*-actin. (b)–(d) Western blot (b) or immunofluorescence analysis ((c) and (d)) indicated *α*-synuclein overexpression in protein level in serially propagated SH-SY5Y-Syn stable cell line; results were normalized to *β*-actin. The GFP-positive cells overexpressed *α*-synuclein. Statistical significance was showed as _ _**P < 0.01, ns: no significance.

Firstly, we established the SH-SY5Y cell line overexpressing *α*-synuclein, SH-SY5Y-Syn. A significantly higher level of *α*-synuclein mRNA was observed in the SH-SY5Y cells (*t*-test, *P* < 0.01) (Figure 1(a)). And the *α*-synuclein expression in protein level was also upregulated in the SH-SY5Y-Syn cells (*t* test, *P* < 0.01) (Figure 1(b)), revealing by western blot analysis, or immunofluorescence analysis (Figure 1(c)). Interestingly, the overexpressed *α*-synuclein did not aggregate in the SH-SY5Y-Syn cells; no aggregated dots were observed under fluorescence microscope (Figure 1(c)). Moreover, the cell line is expressing *α*-synuclein in a stabilized level within five passages (Figures 1(a), 1(b), and 1(d)).

### 3.2. Blockage of Hsp70 by Chemicals Promotes *α*-Synuclein Aggregation in SH-SY5Y-Syn Cells

**Figure 2:**
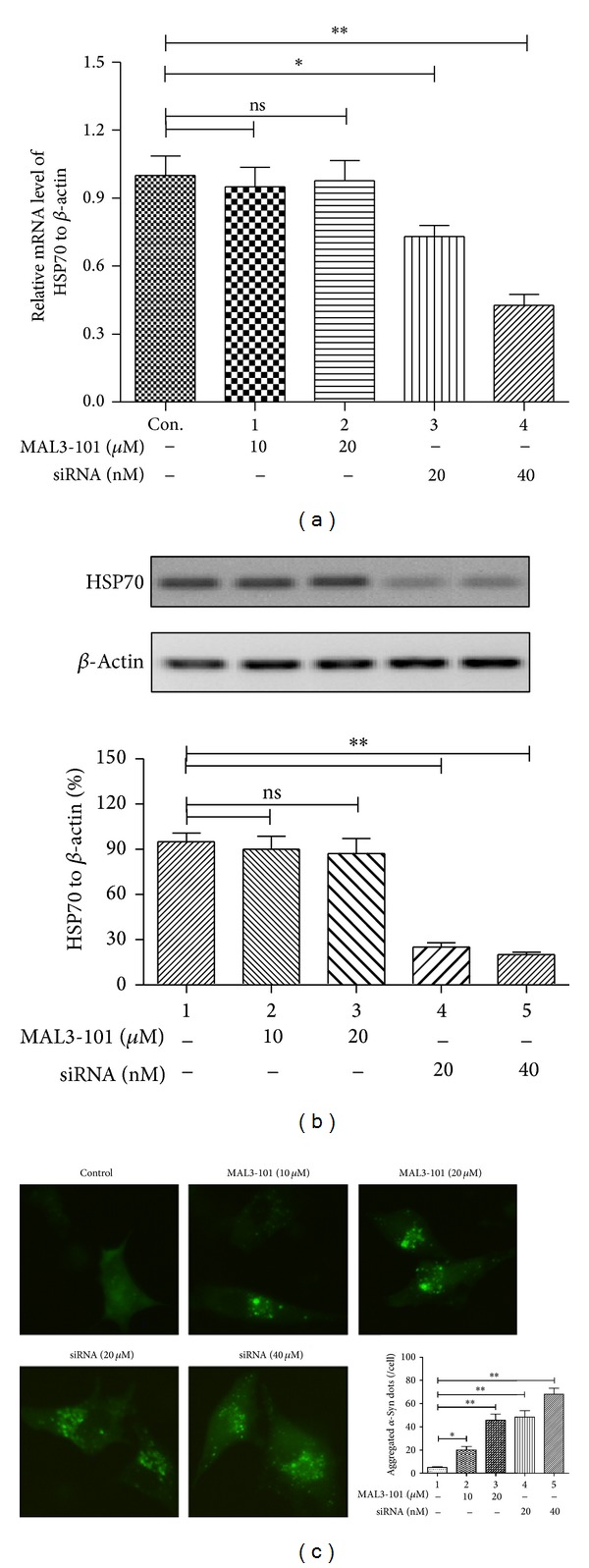
Blockage of Hsp70 by chemicals promotes *α*-synuclein aggregation in SH-SY5Y-Syn cells. (a) Relative Hsp70 expression to *β*-actin in mRNA level, in SH-SY5Y-Syn cells after MAL3-101 treatment or Hsp70 silence by siRNA, revealing by RT-qPCR. (b) Relative Hsp70 expression to *β*-actin in protein level, in SH-SY5Y-Syn cells after MAL3-101 treatment or Hsp70 silence by siRNA, revealing by western blot analysis. (c) Blockage of Hsp70 by chemicals or by RNAi technology promotes *α*-synuclein aggregation in SH-SY5Y-Syn cells. All results were got from triplicate independent experiments. _ _*P < 0.05, _ _**P < 0.01, ns: no significance.

MAL3-101 was used to abrogate the molecular chaperone role of Hsp70. It was shown that the MAL3-101 treatment of 10 or 20 *μ*M had no influence on the Hsp70 expression in both mRNA and protein levels (Figures 2(a) and 2(b)); however, it significantly caused *α*-synuclein aggregation in the SH-SY5Y-Syn cells (Figure 2(c) and Figure 2(d)). And the specific knockdown of Hsp70 by 20 or 40 *μ*M siRNA-Hsp70 transfection (24 hours) led to a significant reduction of Hsp70 expression in both mRNA and protein levels (Figures 2(a) and 2(b)) (*P* < 0.05, or *P* < 0.01) and led to a significant high level of *α*-synuclein aggregation in the SH-SY5Y-Syn cells (Figures 2(c) and 2(d)). Taken together, the Hsp70 abrogation promoted *α*-synuclein aggregation in the SH-SY5Y-Syn cells.

### 3.3. miR-16-1 Targets the 3′ UTR of Hsp70 and Reduces Hsp70 Expression in Human Neuroblastoma SH-SY5Y Cell Line

**Figure 3:**
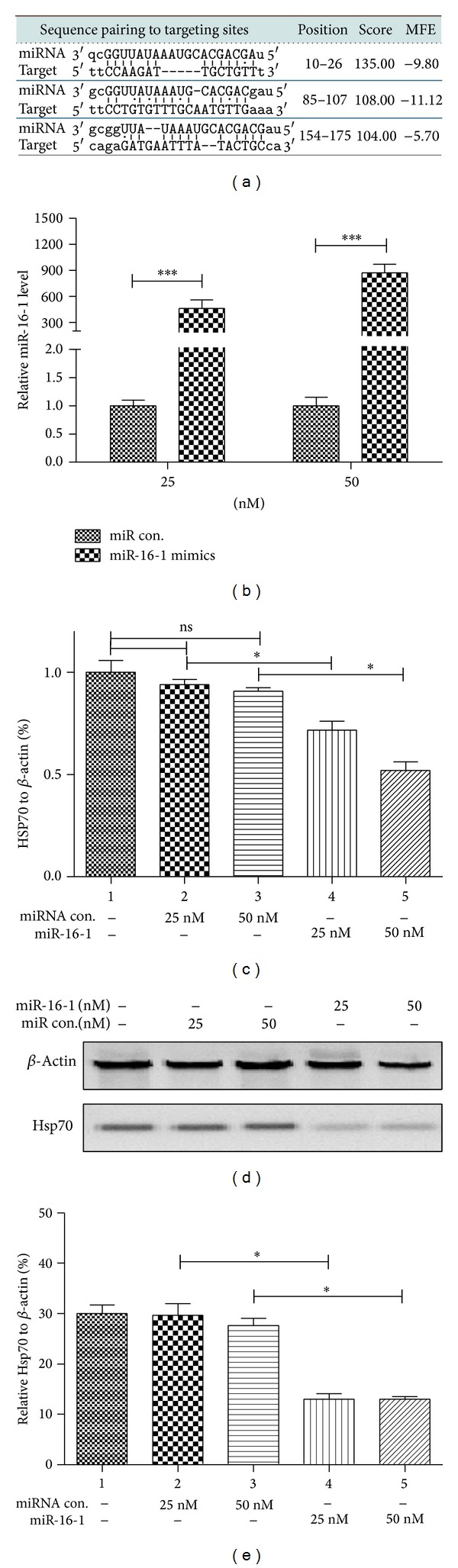
miR-16-1 targets the 3′ UTR of Hsp70 and reduces Hsp70 expression in human neuroblastoma SH-SY5Y-Syn cell line. (a) miR-16-1/3′ UTR of Hsp70 alignment by miRanda analysis. (b) The manipulation of miR-16-1 level in SH-SY5Y-Syn cells. Cells were transfected with 25 or 50 nM miR-16-1 mimics or miR control; 24 h later, the miR-16-1 level was examined by RT-qPCR. (c) and (d) Hsp70 expression was suppressed in mRNA level (c) or in protein level ((d) and (e)) in SH-SY5Y-Syn cells. All experiments were performed in triplicate. No significance, _ _*P < 0.05, or _ _***P < 0.001.

To investigate the possible regulation of microRNAs in Hsp70 expression, we screened the candidate microRNAs targeting Hsp70 by PicTAR and miRanda, and miR-16-1 was on the top list, with three highly paired sites within the 3′ UTR of Hsp70 (Figure 3(a)). Then the miR-16-1 mimics were used to manipulate the miR-16-1 level in SH-SY5Y-Syn cells; Figure 3(b) indicated that the miR-16-1 mimics transfection with 25 or 50 nM significantly drove the miR-16-1 level (*P* < 0.001). What is more, the Hsp70 expression in both mRNA and protein levels was downregulated by miR-16-1 (Figures 3(c)–3(e)).

### 3.4. miR-16-1 Mimics Promoted *α*-Synuclein Aggregation in SH-SY5Y-Syn Cells

**Figure 4:**
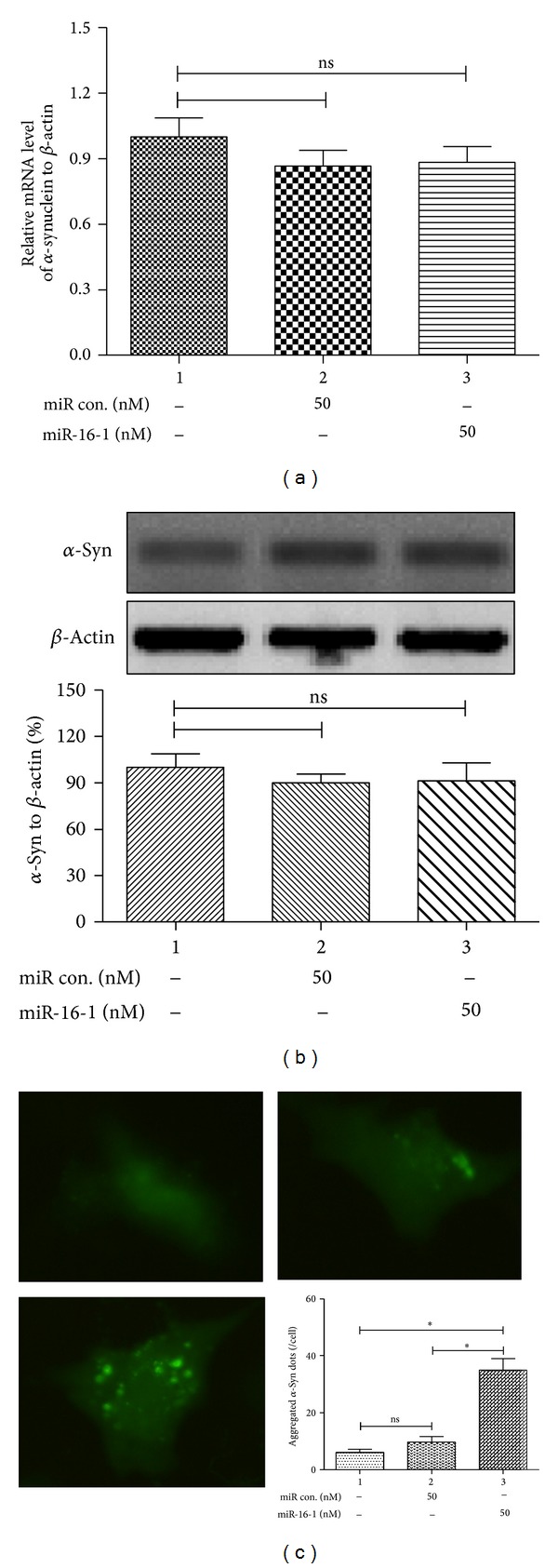
miR-16-1 mimics promoted *α*-synuclein aggregation in SH-SY5Y-Syn cells. (a) and (b) The transfection of miR-16-1 or miR con did not regulate the *α*-synuclein expression in mRNA level (a) or in protein level (b), revealing by RT-qPCR or western blot analysis. (c) miR-16-1 mimics transfection promoted *α*-synuclein aggregation in SH-SY5Y-Syn cells. All results were got from triplicate independent experiments. _ _*P < 0.05 and ns: no significance.

To further explore the regulatory role of miR-16-1 on the *α*-synuclein aggregation, by targeting Hsp70, we transfected SH-SY5Y-Syn cells with 50 nM of miR-16-1 mimics or miRNA control and determined their influence on the *α*-synuclein aggregation. Figures 4(a) and 4(b) indicated that neither 50 nM of miR-16-1 mimics nor 50 nM of miRNA control had influence on the *α*-synuclein expression in mRNA level or in protein level. However, the mimics significantly promoted *α*-synuclein aggregation in the SH-SY5Y-Syn cells (Figure 4(c)) (*P* < 0.05). Thus, we have identified the promotion of miR-16-1 to the *α*-synuclein aggregation in the SH-SY5Y-Syn cells, by targeting and inhibiting the Hsp70 expression.

## 4. Discussion

There is substantial evidence supporting a prominent role in PD-related cell death of amyloid-like aggregation of *α*-Syn [25, 26]. Molecular chaperones are responsible for maintaining protein homeostasis within the cell by assisting protein folding, degradation, and inhibiting protein aggregation [27]. Currently, there is substantial evidence supporting the involvement of dysregulated chaperones, especially Hsp70, in PD pathogenesis [28, 29]. The Hsp70 has been indicated to directly modulate the aggregation and cytotoxicity of *α*-Syn in PD. However, up to now, we have little known about the cause of the Hsp70 failure to maintain the *α*-Syn homeostasis and lead to the *α*-Syn aggregation.

Studies have indicated the important role of microRNAs in the PD development [30–32], such as microRNA-205 [30], mir-34b/c [31], miR-64 and miR-65, and let-7 family [32]. Moreover, several studies have identified the regulatory role of microRNAs in Hsp70 expression, such as miR-378* and miR-711 [33], miRNA-1, miRNA-21, and miRNA-24 [34]. In the present study to investigate the possible regulation of microRNAs in Hsp70 expression and the following *α*-Syn aggregation, we screened the candidate microRNAs against Hsp70, and miR-16-1 was one of the screened target microRNAs, highly pairing sites within the 3′ UTR of Hsp70. The miR-16-1 mimics transfection in SH-SY5Y cell significantly reduced the Hsp70 expression in both mRNA and protein levels in the cell. Along with reported data, the present study has revealed the important regulation of microRNAs in Hsp70 expression.

To evaluate whether there was an influence of Hsp70 downregulation by miR-16-1 on the *α*-Syn aggregation, we firstly constructed an SH-SY5Y cell line overexpressing *α*-synuclein, SH-SY5Y-Syn, in which chemical inhibition of Hsp70 promoted *α*-Syn aggregation. Moreover, the miR-16-1 mimics transfection promoted a significant high level of *α*-synuclein aggregation in the SH-SY5Y-Syn cells, by targeting and inhibiting the Hsp70 expression, though it did not regulate the expression of *α*-synuclein in both mRNA and protein levels.

In summary, we firstly found that miR-16-1 could reduce Hsp70 expression in SH-SY5Y cell line and promote high level of *α*-synuclein aggregation in a *α*-synuclein overexpressed SH-SY5Y cell line.
